# E-cadherin cytoplasmic domain inhibits cell surface localization of endogenous cadherins and fusion of C2C12 myoblasts

**DOI:** 10.1242/bio.013938

**Published:** 2015-10-09

**Authors:** Masayuki Ozawa

**Affiliations:** Department of Biochemistry and Molecular Biology, Graduate School of Medical and Dental Sciences, Kagoshima University, 8-35-1 Sakuragaoka, Kagoshima 890-8544, Japan

**Keywords:** Adhesion, Cadherin, Catenin, Fusion, Myoblast

## Abstract

Myoblast fusion is a highly regulated process that is essential for skeletal muscle formation during muscle development and regeneration in mammals. Much remains to be elucidated about the molecular mechanism of myoblast fusion although cadherins, which are Ca^2+^-dependent cell–cell adhesion molecules, are thought to play a critical role in this process. Mouse myoblasts lacking either N-cadherin or M-cadherin can still fuse to form myotubes, indicating that they have no specific function in this process and may be functionally replaced by either M-cadherin or N-cadherin, respectively. In this study, we show that expressing the E-cadherin cytoplasmic domain ectopically in C2C12 myoblasts inhibits cell surface localization of endogenous M-cadherin and N-cadherin, as well as cell–cell fusion. This domain, however, does not inhibit myoblast differentiation according to microarray-based gene expression analysis. In contrast, expressing a dominant-negative β-catenin mutant ectopically, which suppresses Wnt/β-catenin signaling, did not inhibit cell–cell fusion. Therefore, the E-cadherin cytoplasmic domain inhibits cell–cell fusion by inhibiting cell surface localization of endogenous cadherins and not by inhibiting Wnt/β-catenin signaling.

## Abstract (precis)

**Summary:** This novel approach seeks to overcome the limitations of studying the role of individual cadherins in skeletal muscle fusion *in vitro* by selecting for myoblasts that express dominant-negative E-cadherin, and provides evidence that cadherins play an essential role in myoblast fusion.

## INTRODUCTION

The process by which muscle cells interact and fuse to produce syncytia has been studied for decades and yet remains poorly understood. Myoblasts of different species recognize one another, adhere and fuse to form heterokaryon myotubes, yet very rarely spontaneously fuse with cells from other tissues (Blau et al., 1983). A number of proteins have been implicated in myoblast adhesion and fusion, including N-cadherin, M-cadherin, neural cell adhesion molecule (NCAM), vascular cell adhesion molecule (VCAM-1), meltrin, and various integrins (Dickson et al., 1990; Knudsen et al., 1990; Mege et al., 1992; Menko and Boettiger, 1987; Rosen et al., 1992; Yagami-Hiromasa et al., 1995; Zeschnigk et al., 1995).

Cadherins comprise a large family of Ca^2+^-dependent cell–cell adhesion molecules. E-cadherin, a prototypical member of this family, is a transmembrane protein that forms adherens junctions between epithelial cells. The cytoplasmic domain of cadherins interacts directly with either β-catenin or plakoglobin. These two molecules interact with α-catenin, and α-catenin links the cadherin–catenin complex to the actin cytoskeleton through interactions with α-actinin, vinculin, formin, and actin filaments (Meng and Takeichi, 2009).

Wnt/β-catenin signaling is involved in various aspects of skeletal muscle development and regeneration (Cisternas et al., 2014). The canonical Wnt pathway stabilizes β-catenin and activates target genes via TCF/Lef transcription factors. β-catenin most likely acts as a molecular switch that regulates the transition from cell proliferation to myogenic differentiation (Tanaka et al., 2011). Mice subjected to conditional depletion of β-catenin in the muscle precursor Pax7+ cell lineage (Ctnnb1F/F;Pax7-Cre mice) show reduced muscle mass and fewer slow myofibers (Hutcheson et al., 2009). Mice expressing a constitutively active, stabilized β-catenin in the Pax7+ lineage (Ctnnb**Δ**ex3;Pax7-Cre mice) exhibit reduced myogenesis but more slow myofibers (Hutcheson et al., 2009; Liu et al., 2012). The dysregulation of Wnt/β-catenin signaling can thus lead to severe developmental defects and perturb muscle homeostasis. Blocking Wnt/β-catenin signaling in proliferating cells with an inhibitor decreases proliferation and blocks myoblast fusion during muscle differentiation (Suzuki et al., 2015). Nevertheless, the roles of Wnt/β-catenin signaling during myogenesis remain elusive.

N-cadherin knockout mice die before E10 with disorganized somites, which prevents the analysis of skeletal muscle development (Radice et al., 1997). Explanted, cultured N-cadherin null somites express skeletal muscle specific myosin heavy chain (MHC) but also display normal β-catenin staining at cell–cell contacts (Radice et al., 1997). Similarly, primary N-cadherin null myoblasts can differentiate and fuse normally but express additional classical cadherins, including M-cadherin and cadherin 11 (Charlton et al., 1997). These results indicate that N-cadherin is not essential for myogenesis. Moreover, other cadherins appear to either share redundancy with N-cadherin or compensate for its absence. M-cadherin is also a classical cadherin and is specifically expressed in skeletal muscle and certain neural tissues (Donalies et al., 1991). Numerous observations indicate that M-cadherin is involved in myogenic cell fusion (Zeschnigk et al., 1995; Charrasse et al., 2006). Although these data indicate that M-cadherin is involved in myoblast fusion, mice lacking M-cadherin develop normal skeletal musculature and M-cadherin knockout myogenic cells can fuse normally, suggesting that other molecules can compensate for the lack of M-cadherin *in vivo* (Hollnagel et al., 2002).

The presence of multiple cadherins in myoblasts has hindered the study of their individual roles in skeletal muscle fusion *in vitro*. To overcome these problems, we undertook a novel approach that involves selecting for myoblasts that express dominant-negative E-cadherin. This mutant E-cadherin specifically inhibits the cell surface localization of endogenous cadherins (Ozawa and Kobayashi, 2014). In this report, we show that myoblasts expressing dominant-negative E-cadherin fail to fuse in culture, which provides evidence that cadherins play an essential role in myoblast fusion.

## RESULTS

### Soluble E-cadherin cytoplasmic domain expression in C2C12 cells inhibits cell surface localization of endogenous cadherins

**Fig. 1.**
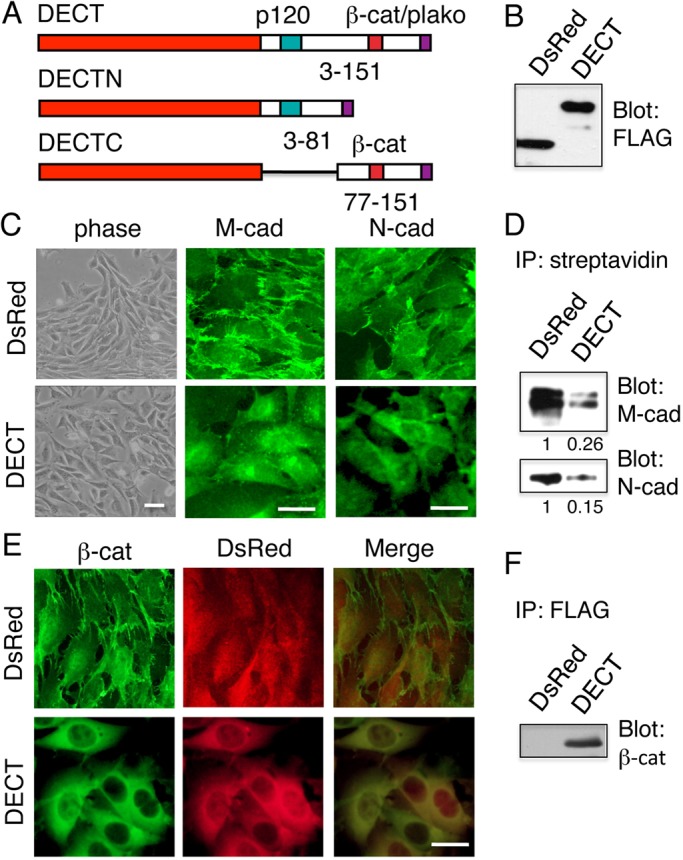
**Expressing DsRed-tagged E-cadherin cytoplasmic domain in C2C12 cells inhibits cell surface localization of endogenous cadherins.** (A) Schematic representation of the DsRed-tagged E-cadherin cytoplasmic domain (DECT) and its derivatives. The green and red boxes indicate the sites that bind p120-catenin and β-catenin (*β-cat*), respectively. DECTN: a chimeric construct composed of DsRed and the N-terminal region of ECT containing the p120-catenin binding site. DECTC: a chimera of DsRed and the C-terminal half of ECT containing the β-catenin binding site. Purple boxes indicate the FLAG tag. (B) Immunoblot detection of FLAG-tagged DsRed or DECT proteins in lysates from C2C12 cells expressing the respective proteins. (C) Morphology and immunostaining of DsRed+ and DECT+ cells with anti-M-cadherin or anti-N-cadherin antibodies. M-cadherin and N-cadherin were detected within intracellular compartments of DECT+ cells. (D) Cell surface proteins were biotinylated as described in the Materials and Methods section. The biotinylated proteins were immunoprecipitated (IP) with streptavidin-agarose. The collected materials were separated on a gel, transferred to a membrane, and subsequently probed with anti-M-cadherin or anti-N-cadherin antibodies. Immunoblot band intensities were quantitated using densitometry, and the relative intensities are indicated below the respective panels. (E) β-catenin co-localizes with DECT but not with DsRed. Cells were stained with antibodies specific for β-catenin. (F) β-catenin co-immunoprecipitates with DECT but not with DsRed. Either DECT or DsRed immunoprecipitated with anti-FLAG and the purified materials were blotted with anti-β-catenin. Scale bars: 25 μm.

We generated a transgene encoding a chimeric construct (DECT) comprising a red fluorescent protein, DsRed, the E-cadherin cytoplasmic domain (ECT), and a C-terminal FLAG epitope tag (Ozawa and Kobayashi, 2014) (Fig. 1A). The *DECT*-coding sequence was expressed under the control of the synthetic CAG promoter in mouse C2C12 myoblasts (Fig. 1B). FLAG-tagged DsRed was used as a control in DsRed+ cells. The inclusion of DsRed facilitated the identification of cells expressing the chimeric protein. Cells expressing DsRed-FLAG ectopically displayed a mesenchymal morphology with predominantly polygonal shapes but were sometimes aligned with their neighbors (Fig. 1B). M-cadherin and N-cadherin were detected at the cell surface (Fig. 1C). In contrast, expressing DECT protein ectopically produced dramatic changes in cultured cells. Cells were loosely associated, and M-cadherin and N-cadherin were detected in intracellular compartments including perinuclear regions (Fig. 1C). DECT expression did not change the distribution of NCAM (data not shown). These results were consistent with our previous finding that DECT expression in epithelial MDCK cells specifically blocks the transport of endogenous E-cadherin but not of gp135, an unrelated cell surface protein (Ozawa and Kobayashi, 2014).

To further determine the localization of M-cadherin and N-cadherin at the outer and inner faces of the plasma membrane, we selectively biotinylated the cell surface of DsRed+ and DECT+ cells with a membrane-impermeable reagent, sulfoNHS-biotin. Biotinylated proteins were isolated with immobilized streptavidin before being subjected to immunoblot analysis for either M-cadherin or N-cadherin (Fig. 1D). Although M-cadherin and N-cadherin were detected at the plasma membrane of DsRed+ cells, significantly reduced amounts of these cadherins were isolated from the plasma membranes of DECT+ cells (Fig. 1D). These results indicate that DECT expression in C2C12 cells reduced the amounts of M-cadherin and N-cadherin on the cell surface. These findings support our immunohistochemical results.

We were unable to detect β-catenin on the surface membrane of DECT+ cells using immunofluorescence staining. Instead, we observed that β-catenin co-localized with DECT within intracellular compartments (Fig. 1E). Although DsRed was detected in the intracellular compartments of DsRed+ cells, it did not change the distribution of β-catenin. The co-localization of β-catenin with DECT suggests that they form a complex. By immunoprecipitating either DECT or DsRed using anti-FLAG, before immunoblotting with anti-β-catenin, we found that β-catenin co-precipitated with DECT but not with DsRed (Fig. 1F). These findings are consistent with our previous observations that DECT interacts with β-catenin, reduces the level of β-catenin associated with endogenous cadherins, and inhibits cell surface localization of endogenous cadherins when expressed in cells (Ozawa and Kobayashi, 2014). These results support our hypothesis that DECT can be used to analyze the role of cadherins in various cellular phenomena in which multiple cadherins are expressed.

### E-cadherin cytoplasmic domain expression inhibits myoblast fusion

**Fig. 2.**
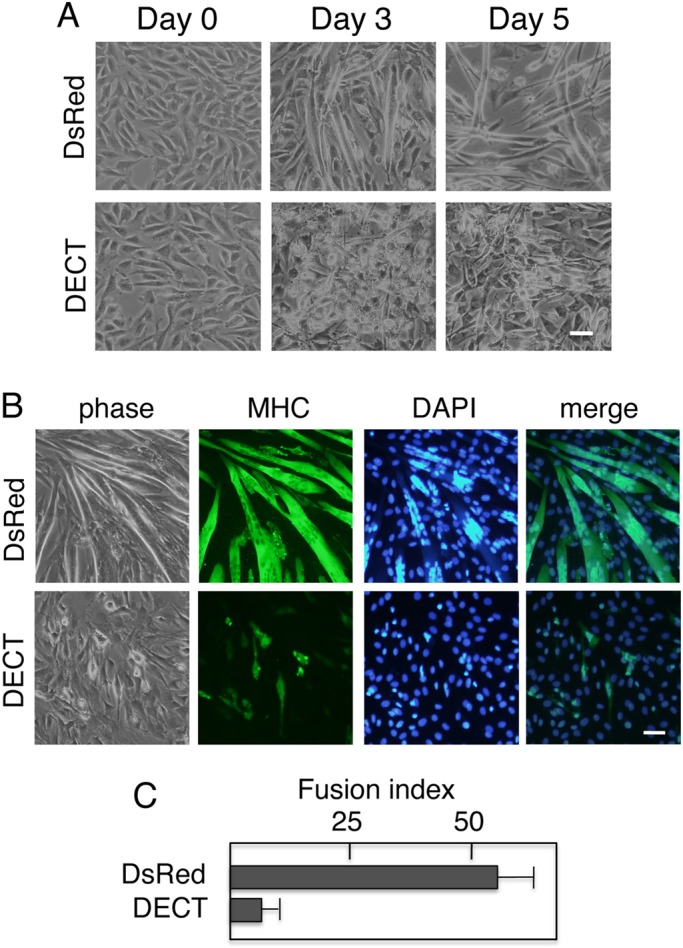
**Expressing E-cadherin cytoplasmic domain inhibits myoblast fusion.** (A) Morphology of C2C12 cells expressing either DsRed or DECT following induction of differentiation. Cells were shifted to differentiation medium the day after plating and analyzed after a further 3 or 5 days. (B) Expression of DECT in C2C12 myoblasts inhibits myoblast fusion. Cells cultured in differentiation medium for 5 days were stained with antibodies specific for myosin heavy chain. Nuclei were counterstained with DAPI. (C) Quantitating myoblast fusion. The fusion index was determined as described in the Materials and Methods section. The results are represented as the mean±s.d. of three independent experiments. Scale bars: 25 µm.

C2C12 cells can be induced to differentiate into muscle cells using low serum culture conditions (differentiation medium) (Yaffe and Saxel, 1977). Under such conditions, control C2C12 cells expressing DsRed generated numerous multinucleated myotubes at days 3 and 5 (Fig. 2A), and the morphological transition from myoblasts to myotubes was also verified by immunohistochemical staining of MHC as a marker for mature muscle cells (Fig. 2B). In contrast to DsRed expression, DECT expression inhibited myotube formation in C2C12 cells (Fig. 2A), and cells that remained unfused and mononuclear were not stained for MHC (Fig. 2B).

The measurement of the fusion index supports our hypothesis that DECT expression inhibits cell fusion and thus myotube formation (Fig. 2C). Almost no myotubes were observed even after 8 days in differentiation medium, indicating that the myoblast to myotube transition was effectively blocked and not just delayed (our unpublished data).

### DECT expression does not affect the induction of myogenin expression

**Fig. 3.**
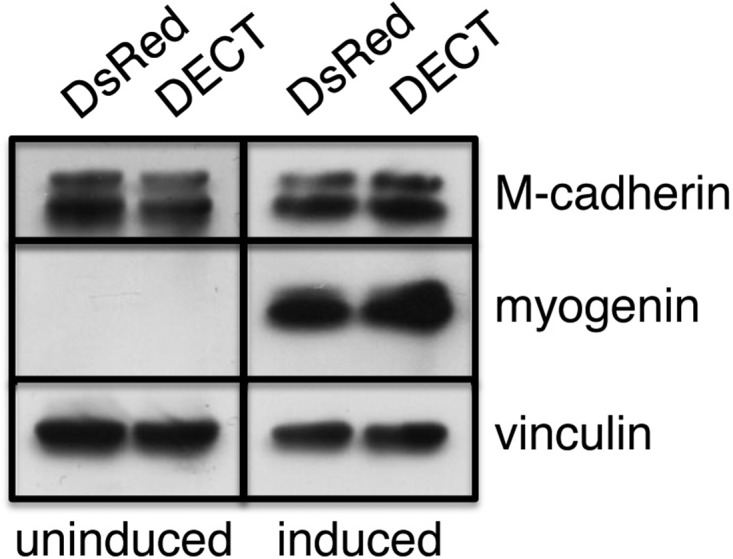
**Immunoblot analysis of differentiation-associated markers.** Cells cultured in differentiation medium for 5 days were subjected to immunoblot analysis with the antibodies indicated on the panel. Vinculin was used as a loading control. Myogenin protein levels were upregulated in both DsRed+ and DECT+ cells.

The differentiation of cultured skeletal myoblasts activated by growth factor withdrawal is accompanied by transcriptional activation of muscle-specific genes, including *MYOD*, *MYF5*, and myogenin (*MYOG*) (Olson, 1992, 1993). These transcription factors orchestrate the entire expression program of the various muscle-specific genes (Olson and Klein, 1994; Yun and Wold, 1996). In established myoblast lines, including C2C12 cells, *MYOD* and *MYF5* are already expressed before differentiation is induced, and myogenin transcription is upregulated upon myogenic induction (Olson and Klein, 1994). Consistent with these observations, microarray analysis of C2C12 cells expressing either DsRed or DECT revealed that *MYOD* and *MYF5* mRNA are already present before differentiation is induced and did not change in level following induction (Table 1). Myogenin mRNA expression was upregulated upon myogenic induction in DsRed+ and DECT+ C2C12 cells (Table 1). As myogenin activity is crucial for activating the entire differentiation program, we determined its protein level using immunoblotting. The expression of myogenin protein (MyoG) was elevated in cultures of both DsRed+ and DECT+ cells, even though the latter rarely fuse into myotubes (Fig. 3). Therefore, DECT expression did not affect the upregulation of myogenin expression following myogenic differentiation. As shown in Table 1, the expression of other differentiation-regulated genes was either upregulated or downregulated upon induction of differentiation in DsRed+ and DECT+ cells.

**Table 1.**
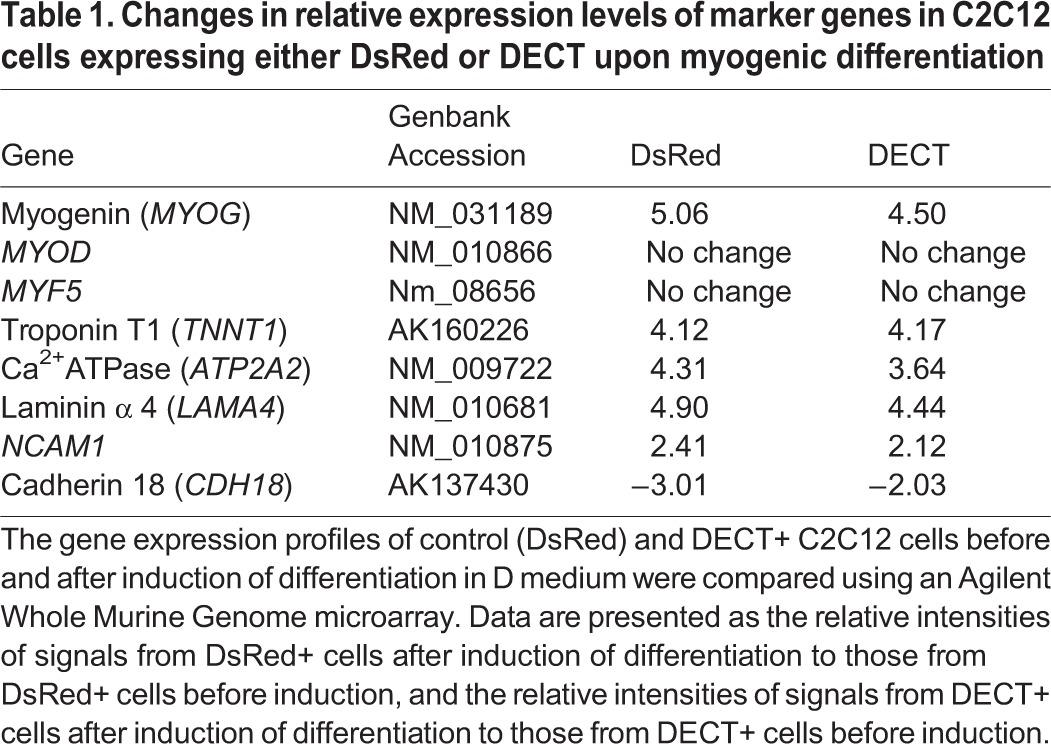
**Changes in relative expression levels of marker genes in C2C12 cells expressing either DsRed or DECT upon myogenic differentiation**

### β-catenin signaling activation during myogenic differentiation

**Fig. 4.**
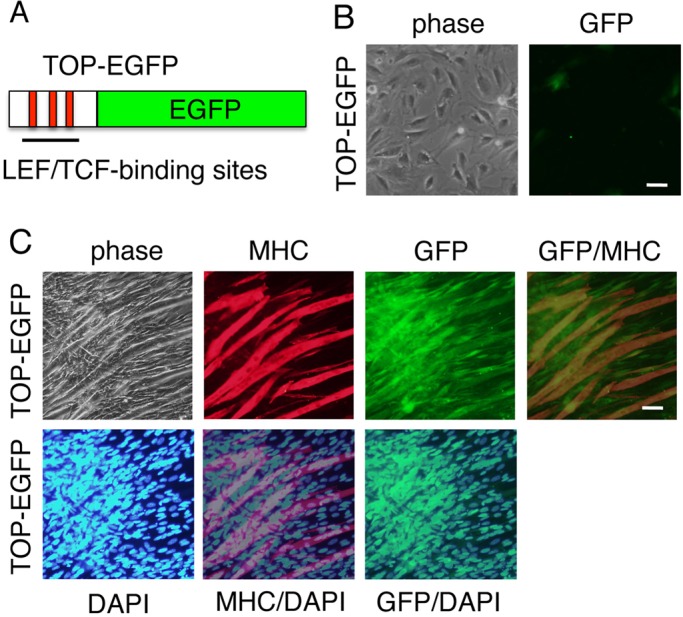
**The Wnt/β-catenin pathway is activated during C2C12 cell differentiation.** (A) Schematic representation of the Wnt/β-catenin pathway reporter construct, TOP-EGFP. The red boxes indicate three LEF-1/TCF-binding sites in the promoter region followed by a green box representing the EGFP coding sequence. (B) A stable C2C12 transfectant harboring TOP-EGFP cultured in GM. The cells show low levels of EGFP expression. (C) Upon induction of differentiation for 5 days in DM, the cells become EGFP-positive. The cells were stained with anti-MHC and DAPI. Scale bars: 25 μm.

The Wnt/β-catenin signaling pathway has been reported to play key roles in myogenic fate determination and differentiation (Cossu and Borello, 1999; Petropoulos and Skerjanc, 2002; Ridgeway et al., 2000). Canonical Wnt signaling is also reportedly involved in myogenic differentiation of mouse myoblasts (Tanaka et al., 2011). Since β-catenin is a critical player in the Wnt signaling pathway (Clevers, 2006), β-catenin sequestration by cadherin's cytoplasmic domain has been shown to block its nuclear translocation and therefore inhibit β-catenin-mediated transcription activity (Sadot et al., 1998; Orsulic et al., 1999; Simcha et al., 2001). Inhibition of β-catenin signaling by DECT may result in the suppression of myogenic cell differentiation and myotube formation. To determine whether β-catenin signaling is activated during C2C12 cell differentiation, we performed a reporter assay using a transgenic construct that expresses EGFP under the control of tandem repeats of a LEF-1/TCF binding site (TOP-EGFP) (Korinek et al., 1997) (Fig. 4A). The same construct was previously used to successfully monitor LEF-1–dependent β-catenin activity (Arnold et al., 2000). The construct was introduced into C2C12 cells and stable transfectants were isolated. When TOP-EGFP+ cells were cultured in growth medium, they exhibited low levels of EGFP protein as determined by immunofluorescence staining (Fig. 4B). When the cells were induced to differentiate under low serum conditions, they expressed high levels of EGFP (Fig. 4C). Although high levels of EGFP were detected in giant multinucleated cells, the area showing a strong EGFP signal did not coincide with the area showing strong MHC staining signal (Fig. 4C), raising the possibility that the Wnt/β-catenin signaling pathway becomes inactivated either during the later stages of differentiation or after myoblast fusion. In any case, activation of the Wnt/β-catenin signaling pathway occurs during the differentiation of C2C12 cells.

### Inhibiting β-catenin signaling is not sufficient to prevent myoblast fusion

**Fig. 5.**
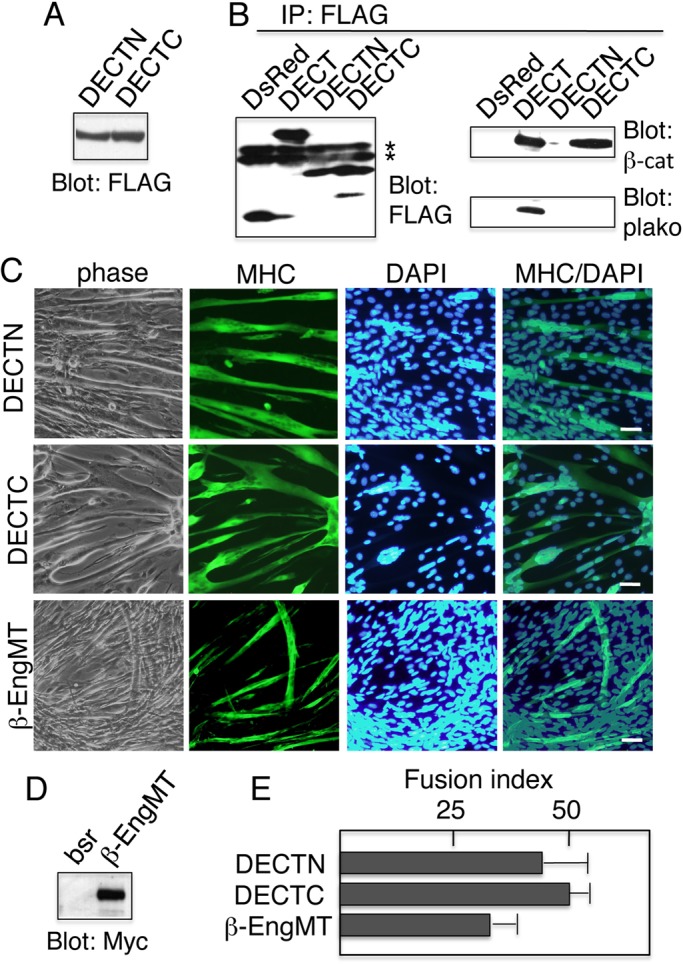
**Inhibiting β-catenin signaling is not sufficient to prevent myoblast fusion.** (A) Immunoblot detection of DECTN or DECTC proteins expressed in C2C12 cells. DECTN and DECTC were detected using anti-FLAG. (B) Immunoprecipitation with anti-FLAG followed by immunoblot analysis with antibodies specific for FLAG, β-catenin, and plakoglobin. Asterisks indicate the position of the immunoglobin heavy chain. β-catenin co-immunoprecipitated with both DECT and DECTC, but not with either DsRed or DECTN. Plakoglobin also interacted with DECT and DECTC. However, reduced amounts of plakoglobin co-immunoprecipitated with DECTC, indicating that DECTC shows weakened interactions compared with DECT. (C) Expressing either DECTN, DECTC, or β-EngMT in C2C12 myoblasts does not inhibit myoblast fusion. Cells cultured in DM for 5 days were stained with anti-myosin heavy chain (MHC). Nuclei were counterstained with DAPI. (D) Immunoblot detection of β-EngMT protein expressed in C2C12 cells. The β-EngMT plasmid was introduced together with a vector containing the blasticidin-resistance gene. Control cells (bsr) were isolated by transfection without the β-EngMT plasmid. β-EngMT was detected using anti-Myc antibody. (E) Quantitating myoblast fusion. The fusion index was determined as described in the Materials and Methods section. The results are represented as the mean±s.d. of three independent experiments. Scale bars: 25 μm.

The cytoplasmic domain of E-cadherin sequesters β-catenin and prevents it from binding to LEF/TCF, thereby inhibiting β-catenin–dependent LEF/TCF transcription activity (Conacci-Sorrell et al., 2003). The N-terminal half of the cytoplasmic domain contains the p120-binding site and the C-terminal half of the domain encodes the β-catenin-binding site. The latter domain and even the shorter (30 amino acid) fragment have been shown to effectively inhibit β-catenin-mediated signaling (Simcha et al., 2001). These domains were independently fused to DsRed to generate two chimeras: the N-terminal (DECTN) and C-terminal (DNCTC) fusion proteins (Fig. 1A). We expressed both chimeras in C2C12 cells (Fig. 5A). Immunoprecipitation with anti-FLAG antibody revealed that although DECT and DECTC co-precipitated with β-catenin, DECT co-precipitated with plakoglobin but not DECTC (Fig. 5B). The result is consistent with our previous observation that the C-terminal half of the E-cadherin cytoplasmic domain, when expressed in MDCK epithelial cells, is able to bind to β-catenin but not to plakoglobin (Ozawa and Kobayashi, 2014). Hence, the construct (DECTC) cannot inhibit cell surface localization of endogenous cadherins (Ozawa and Kobayashi, 2014). Importantly, DECTC expression had no effect on myoblast fusion (Fig. 5C).

The β-catenin-engrailed chimera (β-EngMT) (Montross et al., 2000) was generated by replacing the activation domain of β-catenin with the transcription repression domain of Engrailed. This chimera was shown to specifically repress the transcription activity of β-catenin (Montross et al., 2000). C2C12 cells expressing β-EngMT were established (Fig. 5D), and induced to differentiate by culturing in differentiation medium. Like the control C2C12 cells, these cells formed myotubes (Fig. 5C, bottom panels). Thus, β-catenin inhibition is not sufficient to prevent myoblast fusion. Together, these data strongly suggest that β-catenin–dependent LEF-1 transcription activity is not required for the fusion of C2C12 cells.

### A chimeric E-cadherin–α-catenin molecule that does not require β-catenin for its cell surface transport restores myoblast fusion

**Fig. 6.**
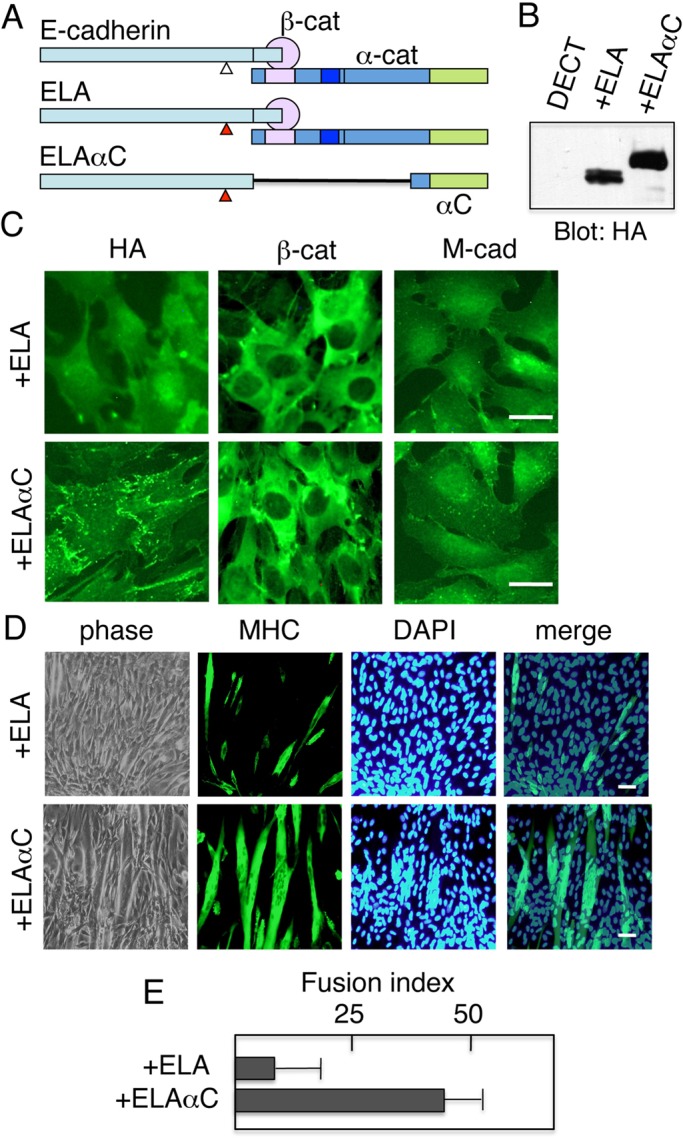
**An E-cadherin–α-catenin chimeric molecule that do not require β-catenin for its cell surface transport restores the ability of myoblast fusion.** (A) Schematic representation of E-cadherin and its derivatives. E-cadherin associates with catenins (α-cat and β-cat). ELA is a mutant E-cadherin in which leucine residues 587 and 588 were substituted with alanines. This substitution improves the cell surface localization of E-cadherin. ELAαC is ELA–α-catenin chimeric protein consisting of (a) the entire extracellular and transmembrane domains of E-cadherin as well as the first 80 amino acids of its cytoplasmic domain, excluding the region required for β-catenin binding, and (b) α-catenin regions encompassing amino acids 612–906, which include the domain necessary for association with ZO-1/actin, but not the domain necessary for association with β-catenin (α-catenin residues 48–163). Thus, ELAαC cannot associate with β-catenin. All constructs were tagged with the HA epitope. (B) Immunoblot detection of ELA, and ELAαC chimera expressed in DECT+ cells. Cell lysates prepared from DECT+ cells and DECT+ cells expressing either ELA (+ELA) or ELAαC (+ELAαC) were analyzed. Blots were stained with anti-HA antibodies. (C) Immunofluorescence staining of DECT+ cells expressing ELA, or ELAαC. Significant amounts of ELAαC, but not ELA, were observed at the cell surface as detected by anti-HA. Expressing either ELA and ELAαC in DECT+ cells did not induce the redistribution of either β-catenin or M-cadherin to the cell surface as these proteins remained in the cytoplasm. (D) Expressing ELAαC, but not ELA, in DECT+ C2C12 myoblasts restores their capacity for myoblast fusion. Cells cultured in DM for 5 days were stained with anti-myosin heavy chain (MHC). Nuclei were counterstained with DAPI. (E) Quantitation of myoblast fusion. The fusion index was determined as described in the Materials and Methods section. The results are represented as the mean±s.d. of three independent experiments. Scale bars: 25 μm.

To confirm that the failure of myoblast fusion was due to disruption of cell–cell junctions by depletion of the β-catenin/plakoglobin required for the correct localization of endogenous cadherins, we performed rescue experiments using chimeric E-cadherin–α-catenin molecules (Fig. 6A). When the chimera, composed of C-terminal truncated E-cadherin and the C-terminal third of the α-catenin polypeptide (residues 612–906, EαC), is expressed in cells, it is transported to the cell surface and is active in aggregation assays (Ozawa, 1998). To improve the chimera's cell surface expression in cells, two leucine residues (587 and 588) in the juxtamembrane cytoplasmic domain were substituted with alanines, yielding ELAαC (Fig. 6A). Leu 587 and 588 are required for the efficient endocytosis of E-cadherin (Miyashita and Ozawa, 2007a) and the intracellular retention of β-catenin–uncoupled E-cadherin (Miyashita and Ozawa, 2007b). We used E-cadherin with the same leucine to alanine (LA) substitutions as a control (Fig. 6A, ELA).

Expression vectors for these constructs, ELA, and ELAαC, were introduced into DECT+ C2C112 cells by transfection. Following selection with blasticidin, stable transfectants were isolated by immunostaining and immunoblotting with anti-HA (Fig. 6B). Although ELA expressed in MDCK cells was localized exclusively to the cell surface (Miyashita and Ozawa, 2007a), the same molecule expressed in DECT+ C2C12 cells was detected in intracellular compartments (Fig. 6C). The unavailability of β-catenin/plakoglobin to complex with ELA, despite the LA substitution, may be responsible for ELA's observed intracellular localization. The E-cadherin–α-catenin (ELAαC) chimera was detected at the cell surface when it was expressed in DECT+ cells (Fig. 6C). This protein cannot interact with β-catenin/plakoglobin because it lacks catenin-binding sites. Therefore, it did not change the subcellular distribution of β-catenin (Fig. 6C). Neither ELA nor ELAαC changed the subcellular distribution of M-cadherin (Fig. 6C).

The cell surface localization of ELAαC in DECT+ cells suggests that cell–cell adhesions are established by expressing ELAαC, despite the presence of DECT, which sequestered β-catenin and prevented the cell surface localization of endogenous cadherins as well as exogenously introduced ELA protein. To determine the capacity for cell fusion, these cells were allowed to differentiate by culturing in differentiation medium. Although ELA expression did not increase the extent of myoblast fusion, ELAαC expression increased the fusion index by almost four times (Fig. 6D and E). This result indicates that DECT+ cells failed to fuse because of a failure to establish proper cell–cell contact, and also that ELAαC expression can restore the capacity for myoblast fusion.

### Cre/loxP site-specific recombination system for controlled DECT expression

**Fig. 7.**
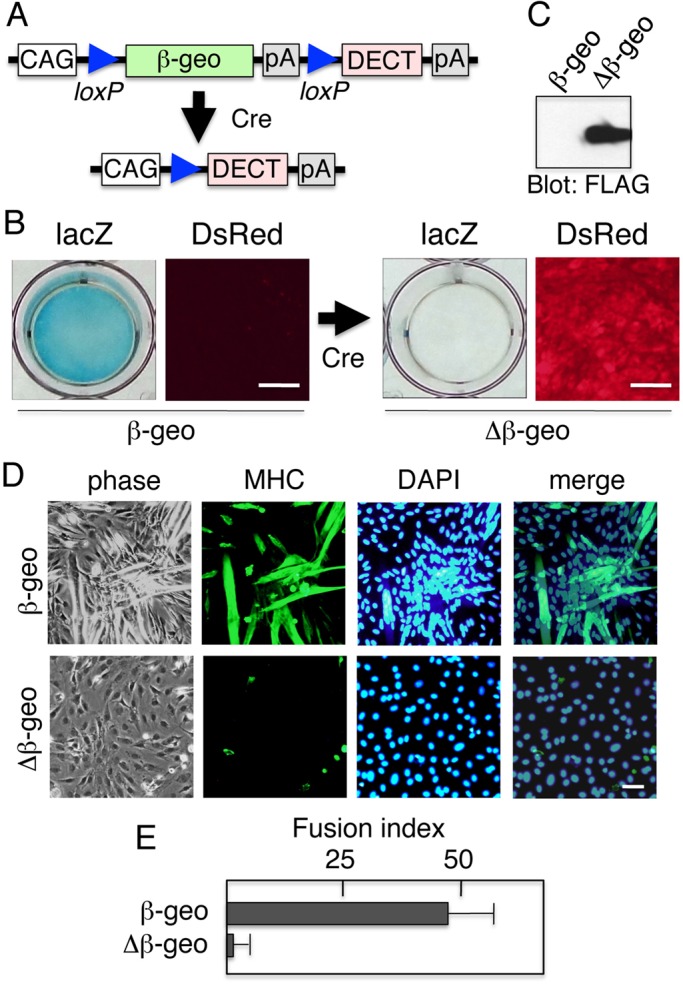
**The Cre/loxP site-specific recombination system for DECT expression.** (A) The *β-geo*/*DECT* expression construct (pCALL-*DECT*) is depicted. The CAG promoter, comprising the CMV enhancer and chicken β-actin promoter, drives the expression of downstream transgenes. The first transgene, *β-geo*, followed by three copies of the SV40 polyadenylation signal (pA) is flanked by loxP sites and is removed by Cre excision. The second transgene, *DECT*, is expressed only after Cre excision, encodes the DsRed-tagged E-cadherin cytoplasmic domain, and is followed by the rabbit β-globin polyadenylation sequence (pA). (B) The *β-geo*/*DECT* expression before and after Cre excision. (Left) *β-geo*/*DECT* C2C12 cells (*β-geo*) were positive for β-galactosidase (LacZ) expression but negative for DECT expression. (Right) *β-geo*/*DECT* C2C12 cells after Cre-mediated excision (Δ*β-geo*) became negative for β-galactosidase activity but at the same time tested positive for *DECT* expression. (C) Immunoblot detection of DECT protein in *β-geo*/*DECT* C2C12 cells before (*β-geo*) or after (Δ*β-geo*) Cre-mediated excision. DECT protein was detected using anti-FLAG. (D) Excision of *β-geo* in *β-geo*/*DECT* C2C12 myoblasts abolishes myoblast fusion. Cells cultured in DM for 5 days were stained with anti-myosin heavy chain (MHC). Nuclei were counterstained with DAPI. (E) Quantitation of myoblast fusion. The fusion index was determined as described in the Materials and Methods section. The results are represented as the mean±s.d. of three independent experiments. Scale bars: 25 µm.

DECT expression alters the cell adhesion of a variety of cells (Ozawa and Kobayashi, 2014), including embryonic stem cells (our unpublished observations). Therefore, it is desirable to express DECT in a strictly controlled fashion for future *in vivo* studies. The Cre/loxP system (Sauer, 1987) has become an important tool for designing post-integrational switch mechanisms for transgenes in mice. We constructed an expression vector that expresses the *lacZ* reporter gene before Cre-mediated excision and expresses DECT following Cre excision, which removes the *lacZ* gene (see Fig. 7A). This vector introduced into C2C12 cells by transfection. A *lacZ*/*neo* (neomycin resistance) fusion gene (*β-geo*; Friedrich and Soriano, 1991) provided both a visual reporter and a drug selection marker for cells carrying the expression construct. The *β-geo* was followed by a triple repeat of the SV40 polyadenylation signal (Lobe et al., 1999) to stop transcription, and the transgene was flanked by loxP sites. The *DECT*-coding sequence followed the loxP-flanked region. *DECT* was expected to not be expressed until after Cre excision of *β-geo* (Fig. 7A). The selected cell clones were tested for β-galactosidase (LacZ) activity and DECT protein expression. These clones were found to be positive for β-galactosidase activity and negative for DsRed fluorescence (Fig. 7B, left panels). After transfection with the Cre expression plasmid, pCAX2-Cre-IRES2-Puro, which contains the bacteriophage P1 *cre* gene driven by the CAG promoter and followed by IRES, as well as the puromycin-resistance gene (*puro*). Transfected cells were selected for puromycin. The selected cell clones were again tested for β-galactosidase and DECT expression. This time, these clones were found to be negative for β-galactosidase activity and positive for DsRed fluorescence (Fig. 7B, right panels). Immunoblot analysis of these clones with anti-FLAG antibody revealed that FLAG-tagged DECT was expressed only after Cre-mediated excision of *β-geo* gene (Fig. 7C). Thus, Cre excision resulted in β-galactosidase expression being replaced by DECT expression.

When these cells were induced to differentiate by culturing in differentiation medium, cells expressing β-galactosidase (*β-geo*) fused to make myotubes but cells expressing DECT (Δ*β-geo*) underwent decreased myoblast fusion (Fig. 7D,E). Thus, *β-geo* excision resulted in DECT expression and the inhibition of myoblast fusion.

## DISCUSSION

In this report, we demonstrated that the expression of DsRed-tagged E-cadherin cytoplasmic domain (DECT) in C2C12 myoblasts inhibits the transport of endogenous cadherins, including N-cadherin and M-cadherin, as well as myoblast fusion. The former findings are consistent with our previous observations that when DECT is expressed in cells, it interacts with β-catenin and plakoglobin, reduces the levels of these proteins associated with endogenous cadherins, and inhibits the cell surface localization of endogenous cadherins. These results support our hypothesis that DECT can be used to analyze the role of cadherins in various cellular phenomena in which multiple cadherins are expressed. Under these circumstances, knocking out or knocking down a single cadherin species is not sufficient to abrogate cadherin function.

Dominant negative cadherin not only inhibits the cell surface localization of endogenous cadherins but also inhibits β-catenin signaling (Simcha et al., 2001). β-catenin is a component of the cadherin–catenin complex and serves as a linking protein at cadherin-mediated adherens junctions. At adherens junctions, transmembrane cadherins bind to the central portion of β-catenin, which contains Armadillo repeats, while actin-bound α-catenin binds to the amino-terminal domain. Similarly, plakoglobin also interacts with cadherins and α-catenin. In addition to its role as a component of the cadherin–catenin adhesion complex, β-catenin plays a critical role in canonical Wnt signaling as it is the central, non-redundant component of the pathway. Both DECT and DECTC have the ability to bind β-catenin and sequester it from LEF-1/TCF transcription factors, thus inhibiting Wnt/β-catenin signaling; however, only DECT expression, and not DECTC expression, inhibits myoblast fusion. Although DECT can bind to β-catenin and plakoglobin, DECTC interacts with β-catenin but its interaction with plakoglobin is significantly reduced. Therefore, DECT can deplete β-catenin and plakoglobin from endogenous cadherins, and consequently inhibit the cell surface localization of endogenous cadherins. In contrast, DECTC cannot bind plakoglobin strongly. Hence, it cannot inhibit the cell surface localization of endogenous cadherins and the formation of cell–cell contacts (Ozawa and Kobayashi, 2014). DECTC, when expressed in C2C12 cells, also failed to bind plakoglobin and did not inhibit cell–cell fusion (Fig. 5). Therefore, the inhibition of myoblast fusion by DECT seems to be due to its ability to inhibit the cell surface localization of endogenous cadherins in C2C12 myoblasts. More importantly, expressing an E-cadherin and α-catenin chimera in C2C12 cells that also express dominant negative cadherin, rescued the cells from the inhibition of myogenesis and induced cell fusion. This E-cadherin–α-catenin chimera functions as a cell–cell adhesion molecule independently of β-catenin/plakoglobin. It seems, therefore, that the inhibition of cadherin function, and not the inhibition of β-catenin signaling, causes myoblast fusion to fail.

To exclude the possible involvement of Wnt/β-catenin signaling in myoblast fusion, we expressed a dominant negative β-catenin mutant, namely the β-catenin/engrailed chimera (Montross et al., 2000). Consistent with the above observation, we found that it has no effect on myoblast fusion. It has previously been shown that the same construct inhibits skeletal muscle development in pluripotent embryonal carcinoma P19 cells (Petropoulos and Skerjanc, 2002). Thus, the nuclear function of β-catenin is essential for skeletal myogenesis in P19 cells. We do not know why this apparent discrepancy exists between these results and ours. It has been shown that β-catenin is required to express transcription factors upstream of *MYOD* in P19 cells (Petropoulos and Skerjanc, 2002). In the absence of Wnt/β-catenin signaling, *MYOD* expression was suppressed and skeletal muscle development was inhibited. In C2C12 cells, *MYOD* and *MYF5* are already expressed even before differentiation is induced (Olson and Klein, 1994). Therefore, Wnt/β-catenin signaling is not necessary to initiate *MYOD* expression in C2C12 cells. We believe that these differences could be the reason for the apparent discrepancy. Recently, it was shown that an inhibitor of Wnt/β-catenin signaling, IWR1-endo, blocks myoblast fusion (Suzuki et al., 2015). Since the inhibitor not only blocks Wnt/β-catenin signaling but also disrupts cadherin/β-catenin/actin complex formation (Suzuki et al., 2015), our observation that the formation of the cadherin–catenin complex is required for myoblast fusion is consistent with the results of these experiments.

M-cadherin is a member of the classical cadherin family and is specifically expressed in skeletal muscle and certain neural tissues (Donalies et al., 1991). Numerous observations indicate that M-cadherin is involved in myogenic cell fusion. For example, synthetic peptides that bind to the extracellular domain of M-cadherin and block homophilic interactions are able to block myoblast fusion in a dose-dependent manner (Zeschnigk et al., 1995). The importance of M-cadherin for the fusion of cultured myoblasts was verified by the RNA interference method (Charrasse et al., 2006). Using RNAi to downregulate M-cadherin expression inhibited cell fusion, whereas upregulation of M-cadherin expression enhanced fusion (Charrasse et al., 2006). Although these data indicate that M-cadherin is involved in myoblast fusion, mice lacking M-cadherin develop normal skeletal musculature. Moreover, M-cadherin knockout myogenic cells can fuse normally, suggesting that other molecules can compensate for the lack of M-cadherin *in vivo* (Hollnagel et al., 2002). Consistent with this hypothesis, we found that E-cadherin/α-catenin chimera expressed in DECT+ C2C12 cells, in which the transport of endogenous M-cadherin and N-cadherin to the cell surface was significantly inhibited, can rescue cells from failed cell fusion and induce proper fusion. These observations suggest that no one specific cadherin is required for myoblast fusion.

DECT expression in cells results in β-catenin and plakoglobin sequestration from endogenous cadherins and inhibition of cadherin cell surface localization, which causes cell dissociation. Therefore it is desirable to express DECT in a strictly controlled fashion in *in vivo* studies. We constructed an expression plasmid for DECT, which expresses the *lacZ* reporter gene before Cre-mediated excision but removes *lacZ* after Cre excision, thereby permitting DECT expression (pCALL-DECT) (see Fig. 7A) and found that it worked well as expected. When this plasmid was introduced into C2C12 cells by transfection, the selected cell clones were positive for β-galactosidase activity and negative for DECT expression before Cre-mediated excision. After the excision, the clones became negative for β-galactosidase activity and positive for DECT expression. Thus, Cre-mediated excision resulted in the expression of DECT in place of β-galactosidase. When these cells were induced to differentiate by culturing in differentiation medium, cells expressing LacZ (*β-geo*) fused to make myotubes but cells expressing DECT (Δ*β-geo*) showed decreased myoblast fusion (Fig. 7D and E). Thus, *β-geo* excision resulted in DECT expression and the inhibition of myoblast fusion. These experiments demonstrated that the system relying on Cre-mediated excision of the *β-geo* reporter prior to DECT expression can be used as a highly controlled method to express DECT, and should be used in future *in vivo* experiments.

## MATERIALS AND METHODS

### Plasmids

The plasmid containing the E-cadherin cytoplasmic domain construct with an N-terminal DsRed tag and a C-terminal FLAG tag (pC-DECT), and the control plasmid containing DsRed with a C-terminal FLAG tag (pC-DsRed) have previously been described (Ozawa and Kobayashi, 2014). pC-DECTN and pC-DECTC, the chimeric constructs composed of DsRed and the N-terminal or C-terminal half of ECT, respectively, have also been previously described (Ozawa and Kobayashi, 2014). The plasmid encoding β-catenin–engrailed chimera (β-EngMT) (Montross et al., 2000) was provided by Pierre D. McCrea, University of Texas MD Anderson Cancer Center.

An expression plasmid for DECT that expresses the *lacZ* reporter gene before Cre-mediated excision but expresses DECT following the removal of *lacZ* by Cre-mediated excision (pCALL-DECT) (see Fig. 6A) was constructed as follows: cDNA encoding DsRed, the E-cadherin cytoplasmic domain, and the FLAG tag in a pC-DECT vector were amplified by PCR using the following primer pairs: 5′-GGTCGCCACCATG GACAA-3′ and 5′-CACGGCCGCTAGACGCCCTTGTCGTC-3′, digested with *Eag*I, and cloned into the *Xho*I and *Not*I sites of the pCALL vector (Lobe et al., 1999). pCAGGSneo, pCAGGGSpur, and pCAGGShyg, which confer G418 resistance, puromycin resistance, and hygromycin resistance, respectively, have previously been described (Ozawa and Kobayashi, 2014). The Cre expression plasmid, pSR016 (pCAX2-Cre-IRES2-Puro), containing the bacteriophage P1 *cre* gene driven by the CAG promoter and followed by IRES (internal ribosome entry site) as well as the puromycin resistance gene, was provided by Rolf Kemler, Max-Planck Institute of Immunobiology and Epigenetics, Freiburg, Germany.

### Cells and transfection

C2C12 cells (provided by Naotoshi Mimura, Osaka University, Japan) were grown in Dulbecco's modified Eagle's medium (DMEM) supplemented with 10% fetal bovine serum (FBS) (referred to as growth medium). Cells were transfected using the calcium phosphate precipitation method, and subjected to selection using either G418 (1 mg/ml), blasticidin (8 μg/ml), puromycin (5 μg/ml), or hygromycin (300 μg/ml). Stable transfectants were identified by fluorescence microscopy combined with immunoblotting, and were isolated as previously described (Ozawa and Kobayashi, 2014). At least three independent clones were selected for each construct to ensure that any observed effects were not due to phenotypic variability introduced by clonal selection. Muscle differentiation was induced, after 24 h in growth medium, by incubating cells in DMEM supplemented with 0.5% FBS (referred to as differentiation medium).

Transfection of C2C12 cells (5×10^6^) with 10 μg *Sca*I–linearized pCALL-DECT was performed with the Amaxa nucleofector system (Amaxa GmbH, Cologne, Germany) as previously described (Ozawa and Kobayashi, 2015). Two days after transfection, cells were incubated in medium containing 100 µg/ml G418 for an additional 5–7 days to isolate drug-resistant colonies. G418-resistant clones were analyzed for their ability to differentiate in differentiation medium, before they were expanded and frozen. The clones were transfected with pSR016 using the calcium phosphate precipitation method, and subjected to selection using puromycin (5 μg/ml). Clones that became positive for DECT expression after Cre introduction were used for further studies. β-galactosidase (LacZ) activity was assayed using a kit, X-Gal Staining (Gelantis, San Diego, CA, USA).

### Antibodies

Mouse monoclonal anti-myogenin and anti-myosin heavy chain (MHC) were obtained from Abcam Japan (Tokyo, Japan) and R&D Systems (Minneapolis, MN, USA), respectively. Mouse monoclonal anti-troponin T and anti-vinculin, along with FITC-labeled phalloidin, were purchased from Sigma-Aldrich Japan (Tokyo, Japan). Rat monoclonal anti-HA and anti-NCAM (MAB310) were purchased from Roche Diagnostics GmbH (Mannheim, Germany) and Merck Millipore (Billerica, MA, USA), respectively. Other mAbs were purchased from Transduction Laboratories (Lexington, KY, USA). All secondary antibodies were obtained from Jackson ImmunoResearch Laboratories (West Grove, PA, USA).

### Immunoblotting

Immunoblot analysis was carried out as previously described (Ozawa and Kobayashi, 2014). Cells were boiled for 5 min in SDS sample buffer. Cellular proteins were subsequently separated by polyacrylamide gel electrophoresis and transferred to a nitrocellulose membrane. Membranes were blocked with 5% non-fat milk before incubating with a primary antibody for either 2 h at room temperature or overnight at 4°C. Primary antibody incubation was followed by incubation with a peroxidase-conjugated secondary antibody. Bound antibody was visualized by enhanced chemiluminescence (ECL; Amersham International, Little Chalfont, UK). Western blot signals were quantitated using the ImageJ software, and relative intensities were calculated after normalization against the corresponding vinculin signals.

### Cell surface biotinylation

Cells (5×10^5^) were incubated twice with 0.5 mg/ml sulfo-NHS-biotin (Pierce Chemical Co., Rockford, IL, USA) at 4°C. Cells were washed with 50 mM NH_4_Cl in PBS at 4°C before they were stripped with 20 mM Tris-HCl (pH 8.0) containing 1% SDS, boiled for 3 min, passed 4–5 times through a 25-G needle, and finally added to nine volumes of 2% Triton X-100. Biotinylated proteins were collected using streptavidin-conjugated beads (Sigma-Aldrich Japan).

### Immunofluorescence and fusion index

Cells were cultured and differentiated in differentiation medium for 5 days. For immunofluorescence microscopy, cells were fixed with paraformaldehyde and permeabilized with 0.2% Triton X-100. Next, they were incubated with primary antibodies and FITC or rhodamine-labeled secondary antibodies as previously described (Ozawa and Kobayashi, 2014). To label nuclei, 4′-6-diamidino-2-phenylindol (DAPI) was used. Cells were analyzed using a fluorescence microscope (Olympus, Tokyo, Japan) equipped with a CD72 camera (Olympus). Either the total number of nuclei or the number of nuclei within MHC-positive myotubes was counted within 10 individual fields per dish. The fusion index was determined by the calculation: Fusion index (%)=(number of nuclei with in MHC-stained myotubes/total number of nuclei)×100. All experiments were performed in triplicate.

### Gene expression microarray and data analysis

Total RNA was prepared from DsRed+ cells, DsRed+ cells allowed to undergo myogenic differentiation for 5 days, DECT+ cells, and DECT+ cells allowed to undergo myogenic differentiation for 5 days. RNA was isolated using TRIzol Reagent (Invitrogen, Carlsbad, CA, USA) and purified as previously described (Ozawa and Kobayashi, 2014). cRNA was amplified and labeled using a Quick Amp Labeling Kit (Agilent Technologies, Santa Clara, CA, USA) and hybridized to a 44K Agilent 60-mer oligomicroarray (Mouse Oligo Microarray Kit). The hybridized microarray slides were scanned using an Agilent scanner. The relative hybridization intensities and background hybridization values were calculated using Agilent Feature Extraction Software (version 9.5.1.1). Microarray data analysis was supported by Cell Innovator (Fukuoka, Japan).

## References

[BIO013938C1] Arnold S. J., Stappert J., Bauer A., Kispert A., Herrmann B. G. and Kemler R. (2000). Brachyury is a target gene of the Wnt/beta-catenin signaling pathway. *Mech. Dev.* 91, 249-258. 10.1016/S0925-4773(99)00309-3 10704849

[BIO013938C2] Blau H. M., Chiu C.-P. and Webster C. (1983). Cytoplasmic activation of human nuclear genes in stable heterocaryons. *Cell* 32, 1171-1180. 10.1016/0092-8674(83)90300-8 6839359

[BIO013938C3] Charlton C. A., Mohler W. A., Radice G. L., Hynes R. O. and Blau H. M. (1997). Fusion competence of myoblasts rendered genetically null for N-cadherin in culture. *J. Cell Biol.* 138, 331-336. 10.1083/jcb.138.2.331 9230075 PMC2138190

[BIO013938C4] Charrasse S., Comunale F., Grumbach Y., Poulat F., Blangy A. and Gauthier-Rouvière C. (2006). RhoA GTPase regulates M-cadherin activity and myoblast fusion. *Mol. Biol. Cell* 17, 749-759. 10.1091/mbc.E05-04-0284 16291866 PMC1356585

[BIO013938C5] Cisternas P., Henriquez J. P., Brandan E. and Inestrosa N. C. (2014). Wnt signaling in skeletal muscle dynamics: myogenesis, neuromuscular synapse and fibrosis. *Mol. Neurobiol.* 49, 574-589. 10.1007/s12035-013-8540-5 24014138

[BIO013938C6] Clevers H. (2006). Wnt/beta-catenin signaling in development and disease. *Cell* 127, 469-480. 10.1016/j.cell.2006.10.018 17081971

[BIO013938C7] Conacci-Sorrell M., Simcha I., Ben-Yedidia T., Blechman J., Savagner P. and Ben-Ze'ev A. (2003). Autoregulation of E-cadherin expression by cadherin-cadherin interactions: the roles of β-catenin signaling, Slug, and MAPK. *J. Cell Biol.* 163, 847-857. 10.1083/jcb.200308162 14623871 PMC2173691

[BIO013938C8] Cossu G. and Borello U. (1999). Wnt signaling and the activation of myogenesis in mammals. *EMBO J.* 18, 6867-6872. 10.1093/emboj/18.24.6867 10601008 PMC1171749

[BIO013938C9] Dickson G., Peck D., Moore S. E., Barton C. H. and Walsh F. S. (1990). Enhanced myogenesis in NCAM-transfected mouse myoblasts. *Nature* 344, 348-351. 10.1038/344348a0 2179732

[BIO013938C10] Donalies M., Cramer M., Ringwald M. and Starzinski-Powitz A. (1991). Expression of M-cadherin, a member of the cadherin multigene family, correlates with differentiation of skeletal muscle cells. *Proc. Natl. Acad. Sci. USA* 88, 8024-8028. 10.1073/pnas.88.18.8024 1840697 PMC52438

[BIO013938C11] Friedrich G. and Soriano P. (1991). Promoter traps in embryonic stem cells: a genetic screen to identify and mutate developmental genes in mice. *Genes Dev.* 5, 1513-1523. 10.1101/gad.5.9.1513 1653172

[BIO013938C12] Hollnagel A., Grund C., Franke W. W. and Arnold H.-H. (2002). The cell adhesion molecule M-cadherin is not essential for muscle development and regeneration. *Mol. Cell. Biol.* 22, 4760-4770. 10.1128/MCB.22.13.4760-4770.2002 12052883 PMC133893

[BIO013938C13] Hutcheson D. A., Zhao J., Merrell A., Haldar M. and Kardon G. (2009). Embryonic and fetal limb myogenic cells are derived from developmentally distinct progenitors and have different requirements for beta-catenin. *Genes Dev.* 23, 997-1013. 10.1101/gad.1769009 19346403 PMC2675868

[BIO013938C14] Knudsen K. A., Myers L. and McElwee S. A. (1990). A role for the Ca2+-dependent adhesion molecule, N-cadherin, in myoblast interaction during myogenesis. *Exp. Cell Res.* 188, 175-184. 10.1016/0014-4827(90)90157-6 2335185

[BIO013938C15] Korinek V., Barker N., Morin P. J., van Wichen D., de Weger R., Kinzler K. W., Vogelstein B. and Clevers H. (1997). Constitutive transcriptional activation by a beta-catenin-Tcf complex in APC−/− colon carcinoma. *Science* 275, 1784-1787. 10.1126/science.275.5307.1784 9065401

[BIO013938C16] Liu Y., Sugiura Y., Wu F., Mi W., Taketo M. M., Cannon S., Carroll T. and Lin W. (2012). beta-Catenin stabilization in skeletal muscles, but not in motor neurons, leads to aberrant motor innervation of the muscle during neuromuscular development in mice. *Dev. Biol.* 366, 255-267. 10.1016/j.ydbio.2012.04.003 22537499 PMC3358465

[BIO013938C17] Lobe C. G., Koop K. E., Kreppner W., Lomeli H., Gertsenstein M. and Nagy A. (1999). Z/AP, a double reporter for cre-mediated recombination. *Dev. Biol.* 208, 281-292. 10.1006/dbio.1999.9209 10191045

[BIO013938C18] Mege R. M., Goudou D., Diaz C., Nicolet M., Garcia L., Geraud G. and Rieger F. (1992). N-cadherin and N-CAM in myoblast fusion: compared localisation and effect of blockade by peptides and antibodies. *J. Cell Sci.* 103, 897-906. 1487503 10.1242/jcs.103.4.897

[BIO013938C19] Meng W. and Takeichi M. (2009). Adherens junction: molecular architecture and regulation. *Cold Spring Harb. Perspect. Biol.* 1, a002899 10.1101/cshperspect.a002899 20457565 PMC2882120

[BIO013938C20] Menko A. S. and Boettiger D. (1987). Occupation of the extracellular matrix receptor, integrin, is a control point for myogenic differentiation. *Cell* 51, 51-57. 10.1016/0092-8674(87)90009-2 3115595

[BIO013938C21] Miyashita Y. and Ozawa M. (2007a). Increased internalization of p120-uncoupled E-cadherin and a requirement for a dileucine motif in the cytoplasmic domain for endocytosis of the protein. *J. Biol. Chem.* 282, 11540-11548. 10.1074/jbc.M608351200 17298950

[BIO013938C22] Miyashita Y. and Ozawa M. (2007b). A dileucine motif in its cytoplasmic domain directs beta-catenin-uncoupled E-cadherin to the lysosome. *J. Cell Sci.* 120, 4395-4406. 10.1242/jcs.03489 18057030

[BIO013938C23] Montross W. T., Ji H. and McCrea P. D. (2000). A beta-catenin/engrailed chimera selectively suppresses Wnt signaling. *J. Cell Sci.* 113, 1759-1770. 10769207 10.1242/jcs.113.10.1759

[BIO013938C24] Olson E. N. (1992). Interplay between proliferation and differentiation within the myogenic lineage. *Dev. Biol.* 154, 261-272. 10.1016/0012-1606(92)90066-P 1330787

[BIO013938C25] Olson E. N. (1993). Signal transduction pathways that regulate skeletal muscle gene expression. *Mol. Endocrinol.* 7, 1369-1378. 8114752 10.1210/mend.7.11.8114752

[BIO013938C26] Olson E. N. and Klein W. H. (1994). bHLH factors in muscle development: dead lines and commitments, what to leave in and what to leave out. *Genes Dev.* 8, 1-8. 10.1101/gad.8.1.1 8288123

[BIO013938C27] Orsulic S., Huber O., Aberle H., Arnold S. and Kemler R. (1999). E-cadherin binding prevents beta-catenin nuclear localization and beta-catenin/LEF-1-mediated transactivation. *J. Cell Sci.* 112, 1237-1245. 10085258 10.1242/jcs.112.8.1237

[BIO013938C28] Ozawa M. (1998). Identification of the region of alpha-catenin that plays an essential role in cadherin-mediated cell adhesion. *J. Biol. Chem.* 273, 29524-29529. 10.1074/jbc.273.45.29524 9792660

[BIO013938C29] Ozawa M. and Kobayashi W. (2014). Cadherin cytoplasmic domains inhibit the cell surface localization of endogenous E-cadherin, blocking desmosome and tight junction formation and inducing cell dissociation. *PLoS ONE* 9, e105313 10.1371/journal.pone.0105313 25121615 PMC4133371

[BIO013938C30] Ozawa M. and Kobayashi W. (2015). Reversibility of the Snail-induced epithelial-mesenchymal transition revealed by the Cre-loxP system. *Biochem. Biophys. Res. Commun.* 458, 608-613. 10.1016/j.bbrc.2015.02.012 25681770

[BIO013938C31] Petropoulos H. and Skerjanc I. S. (2002). Beta-catenin is essential and sufficient for skeletal myogenesis in P19 cells. *J. Biol. Chem.* 277, 15393-15399. 10.1074/jbc.M112141200 11856745

[BIO013938C32] Radice G. L., Rayburn H., Matsunami H., Knudsen K. A., Takeichi M. and Hynes R. O. (1997). Developmental defects in mouse embryos lacking N-cadherin. *Dev. Biol.* 181, 64-78. 10.1006/dbio.1996.8443 9015265

[BIO013938C33] Ridgeway A. G., Petropoulos H., Wilton S. and Skerjanc I. S. (2000). Wnt signaling regulates the function of MyoD and myogenin. *J. Biol. Chem.* 275, 32398-32405. 10.1074/jbc.M004349200 10915791

[BIO013938C34] Rosen G. D., Sanes J. R., LaChance R., Cunningham J. M., Roman J. and Dean D. C. (1992). Roles for the integrin VLA–4 and its counter receptor VCAM-1 in myogenesis. *Cell* 69, 1107-1119. 10.1016/0092-8674(92)90633-N 1377605

[BIO013938C35] Sadot E., Simcha I., Shtutman M., Ben-Ze'ev A. and Geiger B. (1998). Inhibition of β-catenin-mediated transactivation by cadherin derivatives. *Proc. Natl. Acad. Sci. USA* 95, 15339-15344. 10.1073/pnas.95.26.15339 9860970 PMC28044

[BIO013938C36] Sauer B. (1987). Functional expression of the cre-lox site-specific recombination system in the yeast Saccharomyces cerevisiae. *Mol. Cell. Biol.* 7, 2087-2096. 3037344 10.1128/mcb.7.6.2087-2096.1987 PMC365329

[BIO013938C37] Simcha I., Kirkpatrick C., Sadot E., Shtutman M., Polevoy G., Geiger B., Peifer M. and Ben-Ze'ev A. (2001). Cadherin sequences that inhibit beta-catenin signaling: a study in yeast and mammalian cells. *Mol. Biol. Cell* 12, 1177-1188. 10.1091/mbc.12.4.1177 11294915 PMC32295

[BIO013938C38] Suzuki A., Pelikan R. C. and Iwata J. (2015). WNT/β-catenin signaling regulates multiple steps of myogenesis by regulating step-specific targets. *Mol. Cell. Biol.* 35, 1763-1776. 10.1128/MCB.01180-14 25755281 PMC4405648

[BIO013938C39] Tanaka S., Terada K. and Nohno T. (2011). Canonical Wnt signaling is involved in switching from cell proliferation to myogenic differentiation of mouse myoblast cells. *J. Mol. Signal.* 6, 12 10.1186/1750-2187-6-12 21970630 PMC3198762

[BIO013938C40] Yaffe D. and Saxel O. (1977). Serial passaging and differentiation of myogenic cells isolated from dystrophic mouse muscle. *Nature* 270, 725-727. 10.1038/270725a0 563524

[BIO013938C41] Yagami-Hiromasa T., Sato T., Kurisaki T., Kamijo K., Nabeshima Y.-C. and Fujisawa-Sehara A. (1995). A metalloprotease-disintegrin participating in myoblast fusion. *Nature* 377, 652-656. 10.1038/377652a0 7566181

[BIO013938C42] Yun K. and Wold B. (1996). Skeletal muscle determination and differentiation: story of a core regulatory network and its context. *Curr. Opin. Cell Biol.* 8, 877-889. 10.1016/S0955-0674(96)80091-3 8939680

[BIO013938C43] Zeschnigk M., Kozian D., Kuch C., Schmoll M. and Starzinski-Powitz A. (1995). Involvement of M-cadherin in terminal differentiation of skeletal muscle cells. *J. Cell Sci.* 108, 2973-2981. 8537437 10.1242/jcs.108.9.2973

